# Obstructive Sleep Apnea in Women: Study of Speech and Craniofacial Characteristics

**DOI:** 10.2196/mhealth.8238

**Published:** 2017-11-06

**Authors:** Marina Tyan, Fernando Espinoza-Cuadros, Rubén Fernández Pozo, Doroteo Toledano, Eduardo Lopez Gonzalo, Jose Daniel Alcazar Ramirez, Luis Alfonso Hernandez Gomez

**Affiliations:** ^1^ Signal Processing Applications Group Signal, Systems and Radiocommunications Department Universidad Politécnica de Madrid Madrid Spain; ^2^ Audio, Data Intelligence and Speech Group Universidad Autónoma de Madrid Madrid Spain; ^3^ Respiratory Department, Sleep Unit Hospital Quirón Salud de Málaga Málaga Spain

**Keywords:** obstructive sleep apnea, acoustics, speech, image processing, computer-assisted

## Abstract

**Background:**

Obstructive sleep apnea (OSA) is a common sleep disorder characterized by frequent cessation of breathing lasting 10 seconds or longer. The diagnosis of OSA is performed through an expensive procedure, which requires an overnight stay at the hospital. This has led to several proposals based on the analysis of patients’ facial images and speech recordings as an attempt to develop simpler and cheaper methods to diagnose OSA.

**Objective:**

The objective of this study was to analyze possible relationships between OSA and speech and facial features on a female population and whether these possible connections may be affected by the specific clinical characteristics in OSA population and, more specifically, to explore how the connection between OSA and speech and facial features can be affected by gender.

**Methods:**

All the subjects are Spanish subjects suspected to suffer from OSA and referred to a sleep disorders unit. Voice recordings and photographs were collected in a supervised but not highly controlled way, trying to test a scenario close to a realistic clinical practice scenario where OSA is assessed using an app running on a mobile device. Furthermore, clinical variables such as weight, height, age, and cervical perimeter, which are usually reported as predictors of OSA, were also gathered.

Acoustic analysis is centered in sustained vowels. Facial analysis consists of a set of local craniofacial features related to OSA, which were extracted from images after detecting facial landmarks by using the active appearance models. To study the probable OSA connection with speech and craniofacial features, correlations among apnea-hypopnea index (AHI), clinical variables, and acoustic and facial measurements were analyzed.

**Results:**

The results obtained for female population indicate mainly weak correlations (*r* values between .20 and .39). Correlations between AHI, clinical variables, and speech features show the prevalence of formant frequencies over bandwidths, with F2/i/ being the most appropriate formant frequency for OSA prediction in women. Results obtained for male population indicate mainly very weak correlations (*r* values between .01 and .19). In this case, bandwidths prevail over formant frequencies. Correlations between AHI, clinical variables, and craniofacial measurements are very weak.

**Conclusions:**

In accordance with previous studies, some clinical variables are found to be good predictors of OSA. Besides, strong correlations are found between AHI and some clinical variables with speech and facial features. Regarding speech feature, the results show the prevalence of formant frequency F2/i/ over the rest of features for the female population as OSA predictive feature. Although the correlation reported is weak, this study aims to find some traces that could explain the possible connection between OSA and speech in women. In the case of craniofacial measurements, results evidence that some features that can be used for predicting OSA in male patients are not suitable for testing female population.

## Introduction

Sleep disorders are receiving increased attention as a cause of daytime sleepiness, impaired work, and traffic accidents and are associated with hypertension, heart failure, arrhythmia, and diabetes. The most common form of sleep-disordered breathing is the obstructive sleep apnea (OSA) syndrome, and it is characterized by an obstruction of the upper airway (UA) during sleep at the level of the pharynx, yielding partial (hypopnea) or total (apnea) breathing cessation episodes longer than 10 s at a time [1].

The gold standard for the diagnosis of OSA is a full overnight polysomnography (PSG) test [2] performed in an attended laboratory setting. PSG monitors electrophysiologic variables to score sleep stages and detect arousals and cardiorespiratory variables to detect complete (apnea) or near-complete (hypopnea) cessation of airflow. The OSA severity is determined based on the number of apnea and hypopnea episodes per hour of sleep or apnea-hypopnea index (AHI; mild defined as an AHI of 5-15, moderate as 15-30, and severe as ≥30).

However, PSG is expensive and time-consuming, and, furthermore, the recordings are performed in an unfamiliar environment for the patient. Therefore, faster, noninvasive, and less costly alternatives have been proposed for early OSA detection and severity assessment, such as unattended domiciliary sleep studies.

Although overweight and an excess of regional adipose tissue are considered major risk factors for OSA, there are also other interacting elements in OSA pathogenesis, such as craniofacial abnormalities and an altered UA structure, being approached by several studies since the early approaches by means of the analysis of magnetic resonance imaging [3] until the photometry over digital photographs of head [4,5]. Among OSA phenotype-related characteristics are dental occlusion, longer distance between the hyoid bone and the mandibular plane as described by Lowe and coworkers [6], and relaxed pharyngeal soft tissues and large tongue base as described by Schwab and coworkers [3], which generally cause a longer and more collapsible UA. Consequently, abnormal or particular speech in OSA patients may also be expected from the altered structure or function of their UA.

Therefore, several approaches to speech-based OSA detection have been developed since the acoustic perceptive analysis [7,8] until the most recent proposals for using automatic speech-processing techniques in OSA detection [9]. However, most of the previous mentioned publications have only focused on male subjects. To the best of our knowledge, there are no similar studies that concentrated on female OSA patients, and very few publications are available that discuss this issue [10,11].

Consequently, the main purpose of this paper was to study the potential connection between AHI and speech and facial features, focusing on a female population. Furthermore, we have also considered that it might be interesting to compare our results on male versus female patients. In that way, we can observe how the connection between OSA and speech and facial features can be affected by gender.

For an easy interpretation of our results, similar to [12], acoustic analysis is performed by evaluating formant frequencies and bandwidths on sustained phonations of vowel sounds. Facial features are extracted by identifying a set of relevant landmarks on subjects’ images, following also a rather simple procedure similar to the one we presented in [9]. Statistical analysis using correlation coefficients is employed to evaluate the connection between speech and facial features with AHI. To gain a better understanding of this connection, we have used statistical contrasts (Mann-Whitney *U* tests) among OSA severity groups.

## Methods

### Subjects and Recording Procedure

Patients were provided by the Hospital Quirón Salud de Málaga (Spain). The subjects referred for PSG previously reported symptoms of OSA during a preliminary interview with a pneumonologist, such as excessive daytime sleepiness, snoring, choking during sleep, or somnolent driving. By means of this interview, the subjects’ clinical history was obtained, and an exhaustive physical examination focusing on sleep-related symptoms, associated conditions, comorbidities, and anthropometrics measures was conducted and data collected. Subjects’ weight and height were recorded when wearing light clothes. Body mass index (BMI) was calculated as the ratio of body weight (in kg) and the height (in m^2^). Cervical perimeter (in cm) was also measured at the level of cricothyroid membrane. Most of the subjects are from Andalusia (southern Spain). The majority of subjects were white, with the exception of 1 Chinese. Exclusion criteria included subjects with no Andalusian dialect, subjects with a known history of syndromal craniofacial abnormalities, subjects who have had craniofacial surgery, ethnicity, and subjects with excessive facial hair that significantly obscured facial landmarks, as well as subjects with photograph capture errors (eg, inclination, bad position).

**Figure 1:**
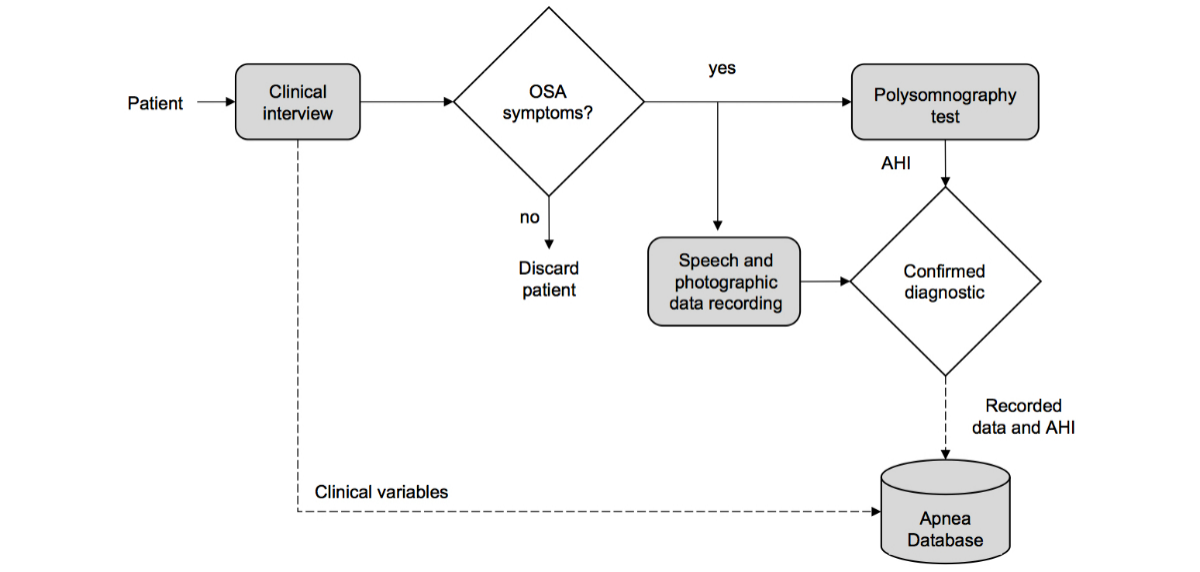
Flowchart of recording data for apnea database. AHI: apnea-hypopnea index; OSA: obstructive sleep apnea.

The diagnosis for each patient was confirmed by specialized medical stuff through standard overnight PSG test, obtaining the AHI on the basis of the number of apnea and hypopnea episodes. According to subjects’ AHI, we defined three groups of OSA severity: low AHI (<10) indicates a healthy subject, AHI between 10 and 30 indicates mild OSA patient, whereas AHI above 30 is associated with severe OSA. These thresholds were defined to get balanced number of samples for our statistical contrast analysis. Figure 1 illustrates the data collection process.

Before the PSG test, all patients were taken to a separate room with adequate acoustic condition and the recording equipment for collecting speech and photographic data, after obtaining patients’ consent. Speech and photographic data are explained as follows:

- Acoustic data: Sustained phonations of each Spanish vowel /a/, /e/, /i/, /o/, and /u/ were recorded from every subject at an upright or seated position and with a comfortable speech level in a quiet room. Recording equipment was a standard laptop computer equipped with an SP500 Plantronics headset microphone. Speech was recorded at a sampling frequency of 50 kHz and encoded in 16 bits. Afterwards, it was downsampled to 16 kHz before processing.
- Photographic data: Frontal and profile digital photographs of the head were obtained before the speech recordings, also at the same normal hospital room without any particular illumination condition. In contrast to the studies by Lee and coworkers [4,5], no special actions were taken beyond a simple control for patients’ front and profile photographs and some instructions to guarantee that the neck area is visible in the profile image. No calibration action for allowing the conversion from pixel measurements to metric dimensions (eg, measuring the distance from the camera) was taken, and manual identification by palpation of facial landmarks was also avoided. A standard Logitech QuickCam Pro 5000 webcam was used to collect images with a size of 640 × 480 pixels and a color depth of 24 bits.

It is important to point out that the recording protocol was approved by the Institutional Review Committee of the Hospital Quirón Salud de Málaga and performed strictly following the ethical consideration of the medical center. The participants were notified about the research and their signed agreement was obtained.

After applying exclusion criteria, a total of 383 subjects (129 women and 254 men) were included in our study. The female population comprised 64 subjects in OSA group (AHI>10) and 65 in control group (AHI≥10). The male population comprised 168 subjects in OSA group (AHI>10) and 86 in control group (AHI≥10). Descriptive statistics of subjects under study are summarized in Table 1.

### Acoustic Features

We focused on formant central frequencies and bandwidths because evidence on the influence of sleep apnea on them has been previously reported by Rob and coworkers [13]. Formants represent resonances of the vocal tract and depend on the UA properties, including its compliance, shape, and dimensions. Hence, these may embed information from specific physiological characteristics in OSA patients, although results shall vary from one sound to another [14]. As mentioned previously, in this contribution, we focused on sustained phonations, which is the common approach for pathologic voices, and apnea may essentially be regarded as one.

Despite these elementary considerations, measuring formant frequencies can be extremely difficult as it is highly influenced by multiple factors, including the method of analysis that is chosen and the analysis settings. Moreover, higher resonances are much more difficult to determine than lower ones because of natural energy losses. Our evaluation on acoustic measurements has shown that, for formants F4 and above, no reliable information could be extracted, and therefore, we restricted our analysis to the first 3 formants. To extract a consistent set of measures on formants’ central frequencies and bandwidths, we followed a specific protocol. First, we computed the values for the first 3 formant central frequencies and bandwidths using 2 different freely available software: the Praat Version 6.0.30 (Praat software, Amsterdam) [15] and the Snack Toolkit Version 2.2.8 (Snack Sound Toolkit, Sweden) [16]. Formant frequencies and bandwidths were estimated every 5 ms using 25-ms long analysis windows. Their values were finally obtained by averaging along the most stable regions of the sustained phonations of each vowel, selecting a steady-state segment of 800 ms where the standard deviation of formant contours was the lowest, excluding initial and ending silences in each utterance.

**Table 1:** Descriptive statistics on Spanish female and male subjects.

Clinical variables	Female (n=129)		Male (n=254)	
	Mean (SD)	Range	Mean (SD)	Range
Apnea-hypopnea index	14.6 (17.0)	0-108.4	22.3 (19.0)	0-87
Weight, in kg	78.0 (18.0)	45-165	92.4 (16.6)	61-162
Height, in cm	161.1 (6.4)	148-178	175.8 (6.9)	160-194
Body mass index, in kg/m^2^	30.1 (6.9)	18.6-63.7	29.9 (4.9)	20.1-52.3
Age, in years	50.9 (11.6)	25-88	48.2 (11.9)	21-78
Cervical perimeter, in cm	36.7 (2.9)	30-45	42.5 (3.2)	34-53

To guarantee a reliable estimation, we measured the absolute differences between estimated values obtained from Praat and Snack for each formant F1-F3. We then manually reviewed those cases for which differences exceeded 70 Hz for F1 and F2 and 150 Hz for F3. These thresholds match the level of accuracy in the reference study by Robb and coworkers [13] and seem consistent with values seen in studies that compare results from Praat with those from Snack [17]. In most cases for which deviations exceeded the prespecified thresholds, one of the two values that had been computed (the one from either Snack or Praat) was found to be incorrect (most often when a formant was skipped). In these cases, the erroneously estimated value was subsequently removed, and the value provided by the other software was retained. In some other cases, both Snack and Praat failed in providing precise results. In those cases, values for formant central frequencies and bandwidths had to be manually selected using spectrograms and linear predictive coding (LPC) analysis. The decision on the number of poles for an optimal fitting of the LPC envelope was based on the general knowledge about the formant structure of each vowel. Values for formants’ central frequencies were obtained as maxima values of the LPC spectral slope, whereas their associated bandwidths were computed by measuring the frequency region around formants’ central frequency within which the spectral envelope amplitude differs −3 dB from the maxima values.

### Facial Features

Facial features were similar than those studied by Lee and coworkers [4,5], including local measurements (ie, areas, distances, angles) extracted from landmarkings on photographs. Major differences in our approach when compared with that of Lee and coworkers [4,5] are the use of supervised automatic image processing and the definition of more robust craniofacial measurements adapted to our less controlled photography capture process.

**Figure 2:**
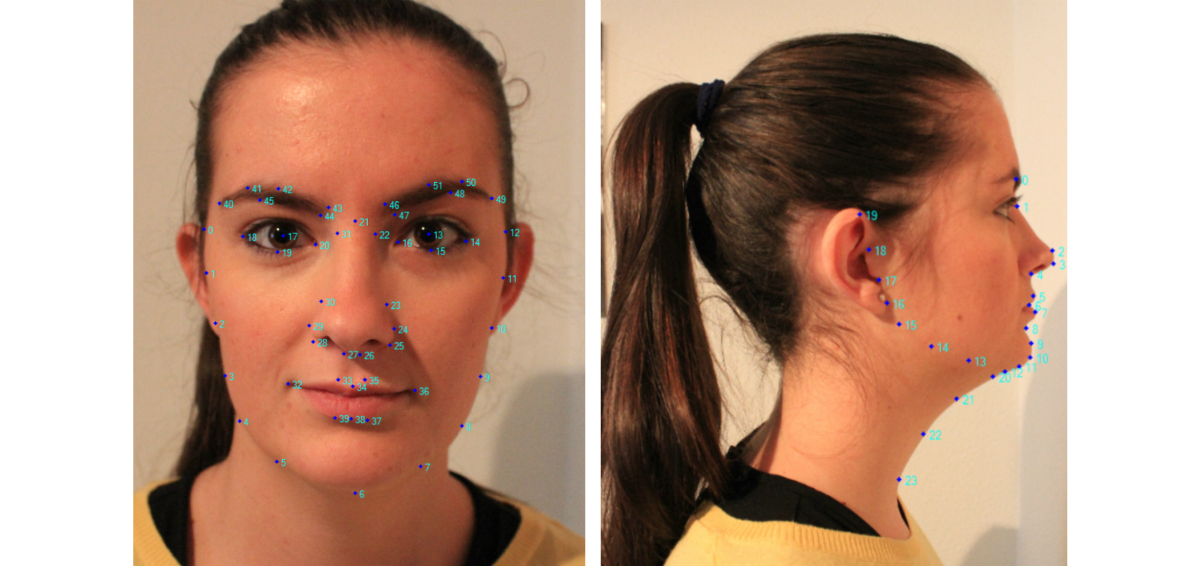
Landmarks on frontal and profile views.

Manual annotation of all images can be tedious, and, even when done by skilled personnel, it is prone to errors because of subjectivity. Consequently, we decided to use a widely used automatic landmarking method, first introduced by Cootes and coworkers [18], based on active appearance model (AAM). On the basis of a priori knowledge of landmark positions, AAM combines a statistical model, which represents the variation of shape and texture of the object, with a gradient-descent fitting algorithm. As depicted in Figure 2, in AAMs for frontal and profile photographs, we used a grid of 52 landmarks taken from a general face identification system and a set of 24 landmarks including specific marks for the neck area, respectively.

During the training stage, frontal and profile AAMs were built from a set of manually annotated photographs using the aam_tools Version 3.0 (aam_tools software, Manchester) [19].

During the fitting stage, starting from a reasonable landmark initialization, the AAM algorithm iteratively corrects the appearance parameters by minimizing the squared error to represent the texture of the target face. Although the AAM performs well for representing shape and appearance variations of an object, the model is location-sensitive to the face’s position. In this study, this effect is increased because photographs were not taken following a highly controlled procedure (illumination conditions, control of distance from the camera, and control of frontal and profile positions). Hence a human-supervised stage was found necessary in order to supervise and, if necessary, correct some large deviations in the automatically generated landmarks.

**Figure 3:**
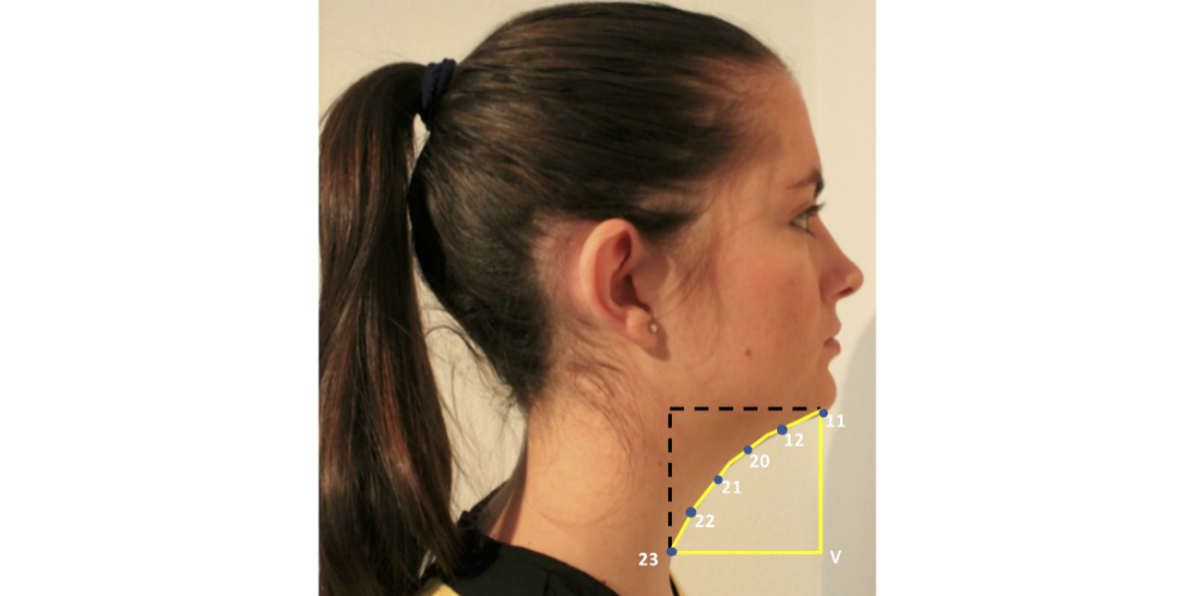
Measurements used for the cervicomental contour ratio.

**Figure 4:**
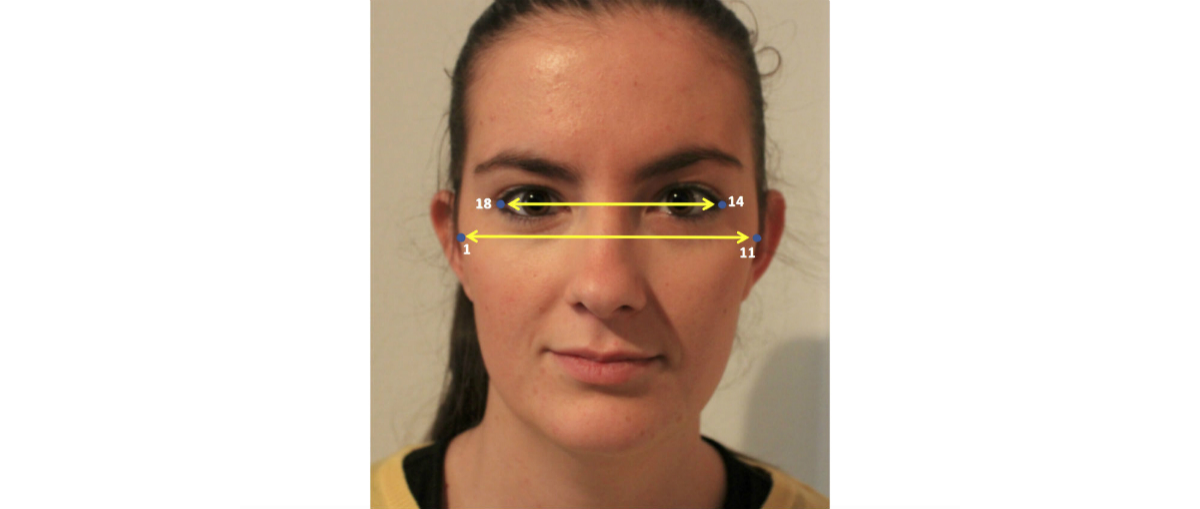
Measurements used for the face-width ratio.

**Figure 5:**
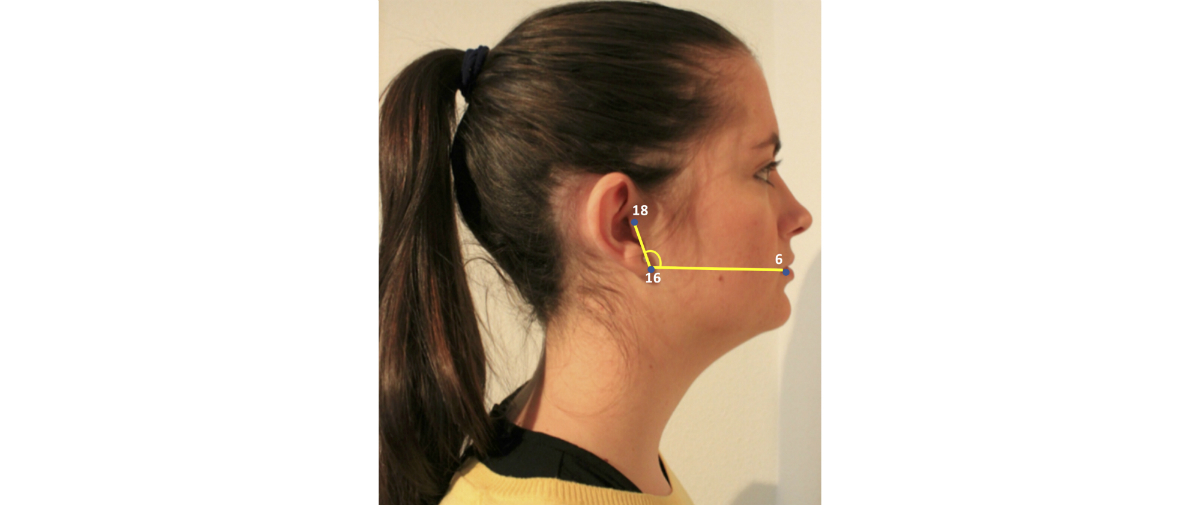
Tragion-ramus-stomion angle.

Once landmarks were generated, we proceeded to extract a set of local features, similar to those studied by Lee and coworkers [4,5] but adapted to our less controlled photographic process. These measurements are described in the following sections:

- Cervicomental contour ratio. One of the anatomical risk factors for OSA is the fat deposition on the anterior neck [20]. This risk factor is captured by a measurement proposed by Lee and coworkers [4,5], that is the cervicomental angle, which is formed by the horizontal plane of the submental region and the vertical plane of the neck. The fat deposition on the anterior neck will cause an increase of this angle. However, considering our limited photography capture process, it is extremely difficult to detect points such as cervical point, thyroid, cricoid, neck plane, or sternal notch involved in the cervicomental region. Consequently, we defined an alternative measurement, more robust to both our image capture and automatic landmarking processes. This measurement was defined using a contour in the cervicomental region traced by 6 landmarks placed equidistantly (ie, landmarks 11, 12, and 20-23 in Figure 3), which were annotated with high reliability following our semiautomatic AAM method. Therefore, the relative measurement of fat deposition on the anterior neck was calculated as the ratio of cervicomental-related area within the rectangular region (ie, yellow solid line defined by landmarks 11, 12, and 20-23, and the bottom right vertex landmark V of the rectangle as depicted in Figure 2) and the area of the rectangular region (ie, black dashed line defined by bottom left landmark 23 and upper right landmark 11 as depicted in Figure 3). This results in an uncalibrated measurement with a value that decreases as the fat deposition on the anterior neck increases.
- Face-width ratio. Lee and coworkers studied the relationship between surface facial dimensions and UA structure in subjects with OSA by means of analysis of magnetic resonance images [21]. Significant positive correlations were detected between surface facial dimensions and UA structures, in particular midface width and interocular width. On the basis of these results, we used these 2 facial dimensions to define a face-width uncalibrated measurement as the midface width to interocular width ratio. The corresponding landmarks and measurements are depicted in Figure 4.
- Tragion-ramus-stomion angle. Lowe and coworkers [6] reported that patients with OSA had retracted mandibles, which is related to the inclination of the occlusal plane and the angle between the relative position of the maxilla to mandible. On the basis of [6], we proposed an uncalibrated measure (ie, an angle) intended to capture, to some extent, the characteristic mandible position or mandibular retraction in OSA individuals. To define this angle, we selected a set of landmarks that not only are related to the posterior displacement of the mandible but also could be accurately detected by our automatic landmarking process on the photographs without need of prior marking. The proposed measurement, as depicted in Figure 5, is the angle between the line ramus-stomion (landmarks 16 and 6) and the ramus-tragion (landmarks 16 and 18).

### Statistical Analysis

To describe our results, we used the strength of the Spearman correlation coefficient as described by Fowler and coworkers [22], that is, values between .01 and .19 are regarded as very weak, .2 and .39 as weak, .40 and .69 as modest, .70 and .89 as strong correlation, and in the range of .90 to .99 as very strong. Values are reported hereafter as mean (SD) and range.

Mann-Whitney *U* test (Wilcoxon rank-sum test) was used to assess significant differences between control and OSA groups because data were not normally distributed.

We conducted our statistical analysis using the Statistic and Machine Learning Toolbox of Matlab.

## Results

Due to the possible effect of clinical variables on correlation between AHI and speech and craniofacial characteristics, we first analyzed the correlation between clinical variables, speech features, craniofacial features, and AHI. Moreover, in order to observe how the connection between OSA and speech can be affected by gender, we also compared correlations between both genders.

### Clinical Variables Analysis

Table 2 presents the Spearman correlation coefficient between clinical variables and AHI for both genders.

As can be seen in Table 2, the strongest correlation for female population found was between age and AHI. Correlations between cervical perimeter, BMI, and AHI are also significant but weak, as well as height, in which case the detected weak correlation is negative. In contrast, in male population, the second strongest correlation found was between weight and AHI, although weak at Fowler scale.

In a comparison by gender, the strongest correlation with AHI is different for each gender: age in the case of women (*r*=.52, *P*=.001) and cervical perimeter in the case of men (*r*=.42, *P*=.001). That is, generally, for both genders, AHI presents significant correlations with age and parameters strongly related to obesity, such as weight, BMI, and cervical perimeter, which are known as risk factors for OSA.

**Table 2:** Spearman correlations between clinical variables and apnea-hypopnea index (AHI) on the female (n=129) and male (n=254) population (for clarity, nonsignificant correlation values are omitted) *.*

Gender	Weight	Height	Age	Cervical perimeter	BMI^a^
Female		−.24^b^	.52^b^	.27^b^	.22^b^
Male	.32^b^		.15^c^	.42^b^	.37^b^

^a^BMI: body mass index.

^b^Correlation is significant at the .01 level (two-tailed).

^c^Correlation is significant at the .05 level (two-tailed).

**Table 3:** Descriptive statistics of the formant frequencies and bandwidth of vowels on the female population (n=129).

Vowel	Formant	Mean (SD)	Range	Bandwidth	Mean (SD)	Range
/a/	F1	859.6 (89.4)	624.4-1070.4	BW1	226.7 (78.7)	57.5-486.5
	F2	1454.0 (105.0)	1174.7-1687.7	BW2	196.6 (82.0)	76-532.6
	F3	2837.2 (221.3)	2186.9-3368.6	BW3	223.2 (97.4)	83.3-576.1
/e/	F1	489.7 (45.8)	377.8-624.3	BW1	98.8 (46.3)	22-266.7
	F2	2268.4 (141.7)	1880.9-2599.9	BW2	143.1 (55.4)	34.0-310.4
	F3	2917.7(159.3)	2597.2-3328.2	BW3	229.8 (81.1)	73.7-473.9
/i/	F1	368.1 (42.2)	243.1-481.5	BW1	68.6 (36.7)	12.2-219.6
	F2	2620.0 (150.9)	2178-2993.2	BW2	131.1 (55.3)	40.3-348.5
	F3	3170.7 (207.3)	2665.6-3645.9	BW3	236.7 (74.7)	86.8-441.4
/o/	F1	537.6 (50.8)	410.4-664.4	BW1	127.7 (61.6)	24.9-360.5
	F2	982.2 (90.5)	758.2-1243.4	BW2	141.2 (67.5)	15.4-395.1
	F3	2881.8 (215.8)	2401.8-3444.9	BW3	155.1 (67.1)	32.9-339.7
/u/	F1	379.3 (49.6)	254.6-509.6	BW1	70.7 (35.6)	8.2-199.2
	F2	823.1 (101.6)	596-1157.9	BW2	152.6 (122.0)	9.0-569.9
	F3	2824.7 (243.1)	2285.7-3790.5	BW3	213.8 (121.5)	53.4-636.2

### Acoustic Features Analysis

Table 3 presents the mean, standard deviation, and the range of formant frequencies and bandwidth for vowels /a/, /e/, /i/, /o/, and /u/ for the female population.

Because of the association between the blockage of the UA and OSA, abnormal or particular speech may be expected in subjects with OSA due to the altered structure of their UA. Likewise, the association between clinical variables (ie, height and weight) and speech [12] is known; thus, indirect association might be expected between speech and OSA. Accordingly, correlations between formant frequencies, bandwidths, and clinical variables are presented in Table 4.

Focusing on formant frequencies, Table 4 shows that the highest, though weak, correlations are found with AHI, age, and cervical perimeter. Surprisingly, none of these formants are correlated with weight, BMI, or height. Moreover, results show that there are 3 formants (F1/a/, F2/e/, and F2/i/), which present weak negative correlation with AHI (*r*=−.26, *P*=.001; *r*=−.24, *P*=.01; *r*=−.26, *P*=.001; respectively). It should be noted that F2/i/ is the only formant correlated with AHI but not correlated with other clinical variables. Likewise, most of the significant correlations with formants were for age, with up to 8 formants. It is known that human voice changes with age [23], which leads us to think that age may cause indirect influence on a relationship between formant frequencies and AHI.

When considering the results for bandwidths in Table 4, only very weak correlations appear: weight negatively correlated with BW1/a/, height with BW3/o/, and age with BW2/a/ and BW2/e/, but no significant correlation was obtained between bandwidths and AHI.

To analyze the gender influence, correlation results in Table 4 were compared with those of a male population, published in our previous study [12] (Table 5). Those results include most of male subjects of the population used in this paper. Given that the difference is very small, we have preferred to use the already published tables with 241 subjects instead of publishing a slightly different one with 254 male subjects.

**Table 4:** Statistically significant Spearman correlation between formant frequencies, bandwidths, and clinical variables on the female (n=129) population (for clarity, nonsignificant correlation values are omitted).

Feature		Apnea-hypopnea index	Weight	Height	Age	Body mass index	Cervical perimeter
**Vowel /a/**							
	Formant, F1	−.26^a^			−.25^a^		−.24^a^
	F2				−.20^b^		
	F3				−.25^a^		−.21^b^
	Bandwidth, BW1		−.19^b^			−.19^b^	
	BW2				−.17^b^		
**Vowel /e/**							
	F2	−.24^a^			−.19^b^		
	F3				−.22^a^		
	BW2				−.21^a^		
**Vowel /i/**							
	F2	−.26^a^					
**Vowel /o/**							
	F2				−.20^b^		−.20^b^
	F3				−.21^b^		−.17^b^
	BW3			−.27^a^			
**Vowel /u/**							
	F2				−.18^b^		

^a^Correlation is significant at the .01 level (two-tailed).

^b^Correlation is significant at the .05 level (two-tailed).

According to Table 5, contrary to the results for the female population, only bandwidths present correlation with AHI—BW2/a/ (*r*=.13, *P*=.05) and BW3/e/ (*r*=−.17, *P*=.01)—but formants do not. The overall results of speech features show that negative correlation coefficients are common between formants, bandwidths, and age. Furthermore, generally those values are smaller (weak at Fowler scale) in both genders.

This finding on the female population showed that 2 of the 3 formant frequencies correlated with AHI also have significant correlation with age (F1/a/ and F2/e/), which leads us to think that age may cause indirect influence on a relationship between formant frequencies and AHI. Similarly, in male population, BW3/e/ is also correlated with weight and BMI, which may indicate an indirect correlation with AHI.

To analyze in detail the influence of each clinical variable on correlation between speech features and OSA, a general review is provided for both genders.

First, we can see that both for male and female populations, most of the significant correlations between acoustic features and clinical variables are linked to age. This is in agreement to several studies on age-related acoustic characteristics, in which different speech features have been reported to correlate with age and have been linked to changes in anatomy and physiology of the speech production system [23]. Some specific studies have reported age-related changes to formants, particularly in the production of vowels. According to these studies, a negative correlation among formants and age, as is also found in our study, can be expected. This lowering of vowel formants with age can presumably be a by-product of the lowering of the vocal folds over the life span in an adult subject, which results in a longer vocal tract [24,25], and with a trend to vowel centralization in older subjects [17,26]. In some cases, these changes have been found to occur only on some particular vowels [25,26]. It should be noted that all the mentioned studies about this issue were performed for both genders.

**Table 5:** Statistically significant Spearman correlation between formant frequencies and clinical variables on the male (n=241) population (for clarity, nonsignificant correlation values are omitted).

Feature		Apnea-hypopnea index	Weight	Height	Age	Body mass index	Cervical perimeter
**Vowel /a/**							
	Formant, F1				−.14^a^		
	F2			−.13^a^	−.13^a^		
	Bandwidth, BW1				−.21^b^		
	BW2	.13^a^					
**Vowel /e/**							
	F1			−.12^a^	−.12		−.17^b^
	F2		−.15^a^	−.20^b^			−.16^a^
	F3			−.21^b^			
	BW1				−.17^b^		−.17^b^
	BW2						−.16^a^
	BW3	−.17^b^	−.14^a^			−.13^a^	
**Vowel /i/**							
	F1			−.15^a^	.16^a^		
	F2			−.21^b^			
	F3						−.20^b^
	BW1				−.13^a^		
	BW3		−.15^a^	−.14^a^			
**Vowel /o/**							
	F1						−.13^a^
	F2				−.27^b^		
	F3			−.17^b^			
	BW1						−.13^a^
	BW2		−.16^a^		.15^a^	−.14^a^	
**Vowel /u/**							
	F1			−.14^a^			
	F2				−.24^b^		
	F3		−.14^a^	−.20^b^			

^a^Correlation is significant at the .05 level (two-tailed).

^b^Correlation is significant at the .01 level (two-tailed).

Considering weight and height, no significant correlations were found for female subjects. These results do not agree with those reported by González [27], where weak and modest correlations with weight and height were found: F2/e/ and height (*r*=−.51), and F2/e/ and weight (*r*=−.50). According to González [27], it seems that the most informative parameters for female height and weight were the second and the third formants from the /a/, /e/, and /i/ vowels. In the case of male population, there are no similarities with that study either. However, unlike women, there are several speech variables with significant correlations with height and weight (see Table 5). In the research by González, stronger correlations were reported for male subjects, mainly between F2/e/ and height (*r*=−.57) and F4/o/ and weight (*r*=−.48), whereas in the OSA male population in [12] the higher correlation coefficient values were obtained between F3/e/, F2/i/, and height (*r*=−.21, *P*=.001, both), and between BW1/a/ and age (*r*=−.21, *P*=.001). Likewise, in case of BMI, no significant correlation with formants was found. One may expect formants’ bandwidths to be larger for OSA patients as an increase in both velar and pharyngeal compliance could result in increased sound damping within the vocal tract [13]. However, only one significant negative correlation was detected between BMI and BW1/a/ (*r*=−.19, *P*=.03) for female patients. A similar situation was found for male patients (BW3/e/ with *r*=−.13, *P*=.05 and BW2/o/ with *r*=−.14, *P*=.03). Despite these clear differences in our studies, both point toward a similar direction: formants seem to be weak predictors of body size in both women and men. Just as in our previous discussion regarding age, it is possible to hypothesize that these significant though weak correlations with height or weight may interfere with specific acoustic characteristics related to OSA.

Finally, cervical perimeter is another feature that is commonly used in discriminating between healthy subjects and OSA patients. More specific than BMI, neck circumference can describe how excessive weight may increase tissue bulk in the neck, which will also increase the dynamic loading of the airway, thus contributing to the pathogenesis of OSA [28]. In the female OSA population under study and similar to what we have found for the other body size measurements, only few significant and weak correlations appeared between cervical perimeter and speech: with F1/a/ and F3/a/ (*r*=−.24, *P*=.01 and *r*=−.21, *P*=.02, respectively), and F2/o/ (*r*=−.20, *P*=.02). Analogous results were found for male subjects (see Table 5). Several previous studies have similarly failed to find modest relationships between voice acoustics and body size effects measured through BMI [28], body mass composition [29] or weight, and neck circumference [30].

### Craniofacial Features Analysis

In this section, descriptive statistics on the female (129) and male (254) subjects under study are shown as well as correlation analysis between craniofacial features and OSA through the AHI. The craniofacial analysis comprises the 3 craniofacial measurements extracted from the landmarks, previously annotated, on patient photographs. Similar to acoustic features, differences by gender were also analyzed. Table 6 presents the mean, SD, and the range of craniofacial measurements. In Table 7, correlations between craniofacial measurements and clinical variables are presented for both genders.

In case of female population, as described in Table 7, all 3 craniofacial measurements present significant but weak correlation with AHI. Cervicomental contour ratio is also modestly correlated with BMI, weight, and cervical perimeter. As regards the face-width ratio, there is a weak negative correlation with height and positive correlation with BMI.

In case of male population, all 3 craniofacial measurements also present correlation with AHI. Furthermore, the strongest correlations are modest, negative, and correspond to BMI, cervical perimeter, and weight, both in men and women.

Furthermore, both genders report significant correlations between all 3 craniofacial measurements and AHI: positive in the case of face-width ratio (*r*=.18, *P*=.04 for women; *r*=.23, *P*=.001 for men), negative for cervicomental contour ratio (*r*=−.23, *P*=.01 for women; *r*=−.37, *P*=.001 for men), and TRG angle (*r*=−.19, *P*=.03 for women; *r*=−.12, *P*=.05 for men).

**Table 6:** Descriptive analysis of the craniofacial measurements on Spanish female and male subjects.

Craniofacial measurements	Female n=129		Male n=254	
	Mean (SD)	Range	Mean (SD)	Range
Cervicomental contour ratio	0.6 (0.1)	0.4-0.9	0.6 (0.09)	0.3-0.8
Face-width ratio	1.4 (0.1)	0.3-1.6	1.5 (0.06)	1.2-1.7
Tragion-ramus-stomion (TRG) angle, in degrees	113.2 (5.7)	97.7-123.7	115.5 (6.22)	98.4-130.4

**Table 7:** Statistically significant Spearman correlation between craniofacial measurements and clinical variables on the female (n=129) and male (n=254) population (for clarity, nonsignificant correlation values are omitted) *.*

Craniofacial measurements		Apnea-hypopnea index	Weight	Height	Age	Cervical perimeter	Body mass index
**Female**							
	Cervicomental contour ratio	−.23^a^	−.65^a^			−.58^a^	−0.66^a^
	Face-width ratio	0.18^b^		−.21^b^			0.22^a^
	Tragion-ramus-stomion (TRG) angle, in degrees	−.19^b^			−.24^a^		
**Male**							
	Cervicomental contour ratio	−.37^a^	−.49^a^		−.17^b^	−.57^a^	−0.59^a^
	Face-width ratio	0.23^a^	0.21^a^			0.17^b^	0.25^a^
	TRG angle, in degrees	−.12^b^					

^a^Correlation is significant at the .01 level (two-tailed).

^b^Correlation is significant at the .05 level (two-tailed).

In general, male population presents stronger values. Indeed, cervicomental contour ratio has the strongest correlation with AHI in both groups. However, as it was pointed out before, this craniofacial measurement also has modest correlation with BMI, weight, and cervical perimeter. Hence, an underlying connection between AHI and cervicomental contour ratio through these clinical variables may exist.

Similar to what was considered for the acoustic feature analysis, we now analyze the influence of each clinical variable on the correlation between craniofacial features and OSA for both genders.

In the case of age, despite the changes in the facial skeleton that occur with aging, only one significant negative correlation between age and craniofacial measurements was found for both men and women: a very weak correlation with cervicomental contour ratio (*r*=−.17, *P*=.01) in the case of male patients and a weak one with TRG angle (*r*=−.24, *P*=.001) in the case of female patients.

As for height, there is only one significant weak correlation with face-width ratio (*r*=−.21, *P*=.02) in female subjects, and no significant correlation was found in male subjects. Regarding this item, there are some controversial conclusions within the scientific community; some of the researches reported strong relationship between craniofacial parameters and stature [31], whereas some of them have not [32,33].

Considering now BMI, weight, and cervical perimeter, in female subjects (Table 7) the more relevant correlations correspond to BMI (*r*=−.66, *P*=.001), weight (*r*=−.65, *P*=.001), and cervical perimeter (*r*=−.58, *P*=.001) with cervicomental contour ratio. In male subjects, higher significant correlations are also related to cervicomental contour ratio with the same clinical parameters (BMI: *r*=−.59, *P*=.001; weight: *r*=−.49, *P*=.001; and cervical perimeter: *r*=−.59, *P*=.001). These results point to cervicomental contour ratio related to the neck and under-the-chin fat depositions as the most likely of facial measurements to be a possible risk factor for OSA.

### Statistical Contrasts Among OSA Severity Groups

In the previous sections, we have studied the correlation between the full AHI range and the set of speech/craniofacial features. In this section, we analyze whether or not these features can be discriminative between two female populations: a control group, defined for AHI<10 and an OSA group for AHI≥10. Similar analyses for male populations were presented by Robb and coworkers in [13] and by ourselves in [12].

Statistical contrasts using Mann-Whitney *U* test among control and OSA groups are presented in Table 8. Looking at the results in Table 8, it can be seen that most of the discriminative speech features reported by Robb are not detected. Only a significant difference in F2/i/ is present, whereas a few novel differences arise for F2/e/, F3 /i/, and BW2/e/.

**Table 8:** Contrast among control and obstructive sleep apnea (OSA) severity groups on the female population (N=129).

Feature		Control (apnea-hypopnea index, AHI <10), n=65	Obstructive sleep apnea (AHI≥10), n=64	*P* value
		Mean (SD)	Mean (SD)	
**Clinical variables**				
	AHI	4.0 (3.1)	25.4 (18.5)	.001^a^
	Weight	76.4 (18.8)	79.7 (17.2)	.31
	Height	162.3 (6.4)	150.9 (6.2)	.04^a^
	Age	45.4 (10.4)	56.5 (10.0)	.001^a^
	Body mass index	29.0 (7.1)	31.2 (6.6)	.04^a^
	Cervical perimeter	36.0 (2.8)	37.5 (2.9)	.01^a^
**Speech features**				
	Formant, F2/e/	2303.5 (129.2)	2232.7 (145.8)	.01^a^
	F2/i/	2664.0 (150.7)	2575.3 (138.5)	.001^a^
	F3/i/	3210.4 (203.5)	3130 (204.9)	.03^a^
	Bandwidth, BW2/e/	152.3 (52.5)	133.9 (57.0)	.02^a^
**Craniofacial features**				
	Cervicomental contour ratio	0.6 (0.1)	0.6 (0.1)	.07
	Face-width ratio	1.4 (0.05)	1.4 (0.06)	.07
	Tragion-ramus-stomion (TRG) angle	114.2 (5.0)	112.0 (6.2)	.06

^a^There are significant differences between OSA groups at the .05 level.

We have reported results for a similar contrast among control and OSA groups for men in [12] (Table 9). This allows us to compare results for female and male populations (Tables 8 and 9, respectively) and see that only F3/i/ appears in both populations. It is also interesting to notice that for males, the remainder significant differences appear only in bandwidths BW1/o/, BW3/o/, BW2/a/, and BW3/e/.

If we analyze now the statistical differences among control and OSA groups for the clinical variables (also shown in Tables 8 and 9), we can see that only weight in females and height in males present no statistical differences. Consequently, it must be concluded the presence of indirect influences of speech and AHI mediated through the rest of clinical variables.

A similar statistical contrast between control and OSA groups was made for craniofacial features. Results showed no significant differences between groups for the female population (see Table 8). Results for our male population are presented in Table 10. These results show significant statistical differences in cervicomental contour ratio and face-width ratio. This points out that the studied facial measurements are more suitable for estimating the AHI in male subjects.

### Matched Groups

As discussed before, our results indicate that there can be an indirect relationship between AHI and both speech and craniofacial features mediated through the clinical variables (age, weight, height, BMI, and cervical perimeter). To evaluate this indirect effect, statistical contrasts are again presented for control and OSA groups but now selected to exhibit no statistical differences among the clinical variables. Thus, the objective was to test whether or not statistical differences previously observed in Tables 8 and 9 (with unmatched values in clinical variables) remain in a matched condition (ie, when there are no statistical differences in clinical variables among control and OSA groups).

Results on matched groups for female population are presented in Table 11, which correspond to control and OSA groups including subjects in the age range of 41 to 55 years and BMI≥25 so that no statistical differences in clinical variables appear.

**Table 9:** Contrast among control and obstructive sleep apnea (OSA) severity groups on a male (N=241) population.

Feature		Control (apnea-hypopnea index, AHI<10), n=81	Mild obstructive sleep apnea, OSA (AHI 10-30), n=87		Severe OSA (AHI>30), n=73	
		Median	Median	*P* value	Median	*P* value
**Clinical variables**						
	Weight	86.0	90.0	.24	99.0	.001^a^
	Body mass index	27.3	28.9	.01^a^	31.4	.001^a^
	Age	42.0	51.0	.001^a^	48.0	.02^a^
	Cervical perimeter	41.0	42.0	.001^a^	44.0	.001^a^
**Speech features**						
	Formant, F3/i/	2707.0	2682.0	.05^a^	2642.0	.03^a^
	Bandwidth, BW1/o/	94.0	79.0	.001^a^	85.0	.10
	BW3/o/	98.0	136.0	.01^a^	107.0	.10
	BW2/a/	118.0	125.0	.08	148.0	.01^a^
	BW3/e/	170.0	201.0	.10	140.0	.03^a^

^a^There are significant differences between OSA groups at the .05 level.

**Table 10:** Contrast among control and obstructive sleep apnea (OSA) groups on the male (N=254) population.

Craniofacial measurements	Control (apnea-hypopnea index, AHI<10), n=86	Obstructive sleep apnea, OSA (AHI≥10), n=168	*P* value
	Mean (SD)	Mean (SD)	
Cervicomental contour ratio	0.6 (0.1)	0.5 (0.1)	.00^a^
Face-width ratio	1.4 (0.1)	1.5 (0.1)	.00^a^
Tragion-ramus-stomion (TRG) angle	116 (6.5)	115.2 (6.1)	.33

^a^There are significant differences between OSA groups at the .05 level.

**Table 11:** Contrast between control and obstructive sleep apnea groups on a subset without differences either on age (41-55 years) or on body mass index (≥25) on the female population.

Feature		Control (apnea-hypopnea index, AHI<10), n=22	Obstructive sleep apnea, OSA (AHI≥10), n=19	*P* value
		Mean (SD)	Mean (SD)	
**Clinical variables**				
	AHI	4.8 (3.0)	26.9 (20.5)	.001^a^
	Weight	86.4 (22.0)	89 (16.4)	.67
	Height	161.2 (6.3)	161.3 (5.1)	.94
	Age	48.9 (4.1)	50.2 (4.2)	.35
	Body mass index	33.3 (8.4)	34.2 (5.9)	.68
	Cervical perimeter	37.1 (2.2)	38 (2.1)	.18
**Speech features**				
	Formant, F2/i/	2670.5 (139.3)	2571.4 (145.8)	.05
**Craniofacial features**				
	Cervicomental contour ratio	0.6 (0.1)	0.6 (0.1)	.67
	Face-width ratio	1.4 (0.04)	1.4 (0.050	.96
	Tragion-ramus-stomion (TRG) angle	114.1 (5.0)	113 (5.1)	.71

^a^There are significant differences between OSA groups at the .05 level.

**Table 12:** Contrast between control and obstructive sleep apnea (OSA) groups on a subset without differences either on age (≤46) or on body mass index (≤30) on the male population.

Feature		Control (apnea-hypopnea index, AHI<10), n=29	Obstructive sleep apnea, OSA (AHI≥10), n=23	*P* value
		Mean (SD)	Mean (SD)	
**Clinical variables**				
	AHI	5.3 (3.0)	27.0 (12.8)	.001^a^
	Weight	87.1 (5.3)	87.1 (8.1)	.99
	Height	178.9 (5.0)	177.6 (5.6)	.35
	Age	38.1 (4.3)	39.0 (5.2)	.47
	Body mass index	27.2 (1.3)	27.5 (1.5)	.35
	Cervical perimeter	40.3 (1.3)	40.7 (1.7)	.38
**Craniofacial features**				
	Cervicomental contour ratio	0.6 (0.07)	0.6 (0.1)	.02^a^
	Face-width ratio	1.4 (0.06)	1.5 (0.05)	.23
	Tragion-ramus-stomion (TRG) angle	116.2 (6.8)	115.7 (5.6)	.73

^a^There are significant differences between OSA groups at the .05 level.

As Table 11 illustrates, once the possible effect of age and BMI is minimized, only significant difference in F2/i/ remains, whereas the differences for F2/e/, F3/i/, and BW2/e/ disappear. This result is coherent with correlations in Table 4, where it can be noted that F2/i/ is only correlated with AHI, whereas correlation with some clinical variables appear for F2/e/ and BW2/e/. Also by comparing Table 8 with Table 11, it can be observed that, as it is reasonable, there are no significant differences in craniofacial measurements in both tables.

Matched results for the male population selecting individuals with age ≤46 and BMI in the range of 25 to 30 are presented in Table 12. Results in this table indicate that only the significant difference in cervicomental contour ratio remains, which indicates that the neck fat deposition is a possible risk factor for OSA in male population, as it was pointed before because higher significant correlation was related to this craniofacial feature (see Table 7).

## Discussion

### Principal Findings

The results of this investigation indicate that acoustic and facial measurements in a female population have weaker correlation with AHI than with clinical variables. Significant correlations for female individuals (mainly weak correlations) are somewhat stronger than those for male subjects (mainly very weak).

In the studied female population, formant frequencies seem to prevail over bandwidths. Specifically, F2/i/ is the speech variable that showed to be a good predictor of OSA syndrome, as it is the only acoustic measurement that remains after contrasting OSA and non-OSA individuals, both unmatched (Table 8) and matched (Table 11), with clinical variables. Regarding craniofacial parameters, according to the results, the particular facial features that we have studied are not suitable to distinguish between OSA and non-OSA female subjects.

In the case of male population, bandwidths seem to prevail in their correlation to AHI over formant frequencies. BW2/a/ and BW3/e/ are the only ones that remain after the same contrast analysis using groups matched in clinical variables (Table 13). Considering craniofacial measurements, cervicomental contour ratio is the variable that is still present after the contrast analysis using matched groups (see Table 12). This outcome suggests that the use of craniofacial measurements is more appropriate to differentiate OSA-affected male patients.

### Limitations

We are aware that our research has several limitations. The first one is that the results presented in this study are limited to Spanish subjects, most of whom are speakers of a single Spanish dialect, the Andalusian. Consequently, a cross-language comparison should be made. Another limitation is that measuring formant frequencies and bandwidths is technically problematic, and it always achieves limited precision. To obtain results with higher accuracy and reliability, future studies will need to examine possible impact of different factors, such as patient’s position or time of the day, during the data collection process, as acoustic differences may be expected.

With regard to craniofacial features, we have only explored uncalibrated craniofacial measurements because we have limited our study to simulate an OSA assessment app running on a mobile device. Our research may also be limited by the precision of the measurements, particularly in the case of the craniofacial measurements.

### Conclusions

An important outcome of our investigation is that there may be a possible underlying impact of clinical variables on the correlations between voice features and OSA. Thus, future research should consider new speech analysis techniques capable of properly compensating unwanted variability due to clinical variables. In the case of craniofacial measurements, the results suggest that the features used in this study are more suitable for male patients than for female patients. Therefore, searching for those specific features that are more convenient for female subjects would be interesting to try to improve the assessment techniques of OSA in women.

Moreover, besides the known OSA risk factors, there are other disorders that can cause OSA, such as hypothyroidism [34] and acromegaly [35] disorders, which can give different craniofacial representation. Comparing these features in different groups could skew the data. Therefore, future studies should also contemplate these related OSA conditions as exclusion criteria to avoid false discoveries.

**Table 13:** Contrast between control and obstructive sleep apnea (OSA) groups on a subset without differences either on age or on height on the male population.

Features		Control (apnea-hypopnea index, AHI<10), n=73	Obstructive sleep apnea, OSA (AHI>30), n=65	*P* value
		Median (SD)	Median (SD)	
**Clinical variables**				
	Weight	85 (11.1)	99 (22.65)	.001^a^
	Height	174 (7.26)	176 (6.61)	.24
	Body mass index	27 (3.79)	32 (6.63)	.001
	Age	43 (9.51)	47 (8.55)	.21
	Cervical perimeter	41 (2.46)	44 (3.7)	.001^a^
**Speech features**				
	Bandwidth, BW2/a/	116 (40.31)	148 (129.4)	.001^a^
	BW3/e/	161 (98.23)	137 (94.6)	.03^a^

^a^There are significant differences between OSA groups at the .05 level.

## Abbreviations

AAM: active appearance model
AHI: apnea-hypopnea index
BMI: body mass index
BW: bandwidth
F: formant
LPC: linear predictive coding
OSA: obstructive sleep apnea
PSG: polysomnography
TRG: tragion-ramus-stomion
UA: upper airway
